# The experience of women infected by the COVID-19 during pregnancy in Brazil: a qualitative study protocol

**DOI:** 10.1186/s12978-020-00958-z

**Published:** 2020-07-08

**Authors:** Juliana Vasconcellos Freitas-Jesus, Larissa Rodrigues, Fernanda Garanhani Surita

**Affiliations:** 1 Postgraduate Program in Obstetrics and Gynecology, School of Medical Sciences, University of Campinas, Campinas, Brazil; 2 Department of Obstetrics and Gynecology, School of Medical Science, University of Campinas, Av. Alexander Fleming, 101, Campinas, SP Brazil

**Keywords:** COVID-19, Coronavirus, Pandemic, Pregnant women, qualitative study, Feelings, Psychology, Social media, Family relations, Community relations

## Abstract

**Background:**

The Coronavirus disease (COVID-19) is highly infectious, with the recent World Health Organization decree confirming a global public health emergency. The outcomes related to maternal and fetal health among pregnant women infected with the virus are still poorly understood. The world population has been waiting for answers and remains constantly alert about the pandemic’s progress. It is not yet known what impact this pandemic experience will have on the population’s mental health, especially pregnant women.

**Method:**

We aim to understand and discuss the experiences of women who were infected by COVID-19 during pregnancy, in relation to the illness process, community relations, and social media influences. This is a qualitative study in which we will interview women who were infected by COVID-19 during pregnancy and received medical care from a tertiary university hospital specializing in women’s health in Brazil. We will use the techniques of Semi-Directed Interviews of Open and In-depth Questions, socio-demographic and health data sheets, and Field Diaries. We will use purposive sampling and the criterion of theoretical saturation for its construction. The interviews will be conducted by phone or video call, with audio recorded for later transcription. The treatment of the data will be completed through Thematic Analysis and discussed in light of the Health Psychology framework, with the production of categories that answer the proposed research questions.

**Discussion:**

It is expected that the results contribute to the understanding about the demands that come to the health professional of women infected by COVID-19 during pregnancy in a pandemic situation.

**Resumo em Português (Portuguese abstract):**

**Introdução:**

A doença causada pelo coronavírus (COVID-19) é altamente infecciosa, com a recente declaração da Organização Mundial de Saúde confirmando emergência global de saúde pública. Os desfechos relacionados a saúde materno-fetal entre gestantes infectadas pelo vírus ainda são pouco conhecidos. A população mundial tem aguardado respostas e se mantém constantemente em alerta sobre o progresso da pandemia. Ainda não se sabe qual será o impacto da experiência da pandemia sobre a saúde mental da população, especialmente entre mulheres grávidas.

**Método:**

O objetivo deste estudo é compreender e discutir as experiências de mulheres infectadas pelo COVID-19 durante a gravidez, em relação ao processo de adoecimento, às relações comunitárias e a influência de mídias sociais. Este é um estudo qualitativo em que serão entrevistadas mulheres infectadas pelo COVID-19 durante a gestação, atendidas em um hospital universitário terciário especializado em saúde da mulher no Brasil. Serão usadas as técnicas de Entrevistas Semi-Dirigidas de Perguntas Abertas em Profundidade, ficha de dados sociodemográficos e de saúde e diários de campo. A amostra será selecionada intencionalmente, usando o critério de saturação teórica para a sua construção. As entrevistas serão conduzidas por telefone ou videoconferência, com áudio gravado para posterior transcrição. O tratamento dos dados seguirá a Análise Temática e os resultados serão discutidos sob conceitos de Psicologia da Saúde, com a produção de categorias que respondam as questões de pesquisa propostas.

**Discussão:**

Espera-se que os resultados contribuam para a compreensão de demandas emergentes entre profissionais de saúde para mulheres infectadas pelo COVID-19 durante a gestação em situação de pandemia.

## Plain English summary

The Coronavirus disease (COVID-19) is highly infectious. The World Health Organization recently decreed a global public health emergency. Scientists are still investigating, but we still know little about the outcomes of maternal and fetal health of pregnant women infected with the virus. The world population has been waiting for answers and remains constantly alert about the pandemic’s progress. It is not yet known what impact this pandemic experience will have on the population’s mental health, especially pregnant women. We will conduct a research project and pregnant women during the next months.

We aim to understand and discuss the experiences of women who were infected by COVID-19 during pregnancy, in relation to the illness process, community relations, and the social media influences. We will interview women who were infected by COVID-19 during pregnancy and received medical care from a tertiary university hospital specializing in women’s health in Brazil. We will use Interviews and In-depth Questions, socio-demographic and health data sheets, and Field Diaries. The interviews will be conducted by phone or video call, with audio being recorded for later transcription. The data will be analyzed systematically, using specific and well stablished methods to do so. We will produce a textual report with categories that answer the proposed research questions.

We believe the results will contribute to the health, professional, and political considerations of managing mental health demands coming from the women infected by COVID-19 during pregnancy in a pandemic situation.

## Background

The severe acute respiratory syndrome–novel coronavirus-2 (SARS-CoV-2), also known as COVID-19, has been considered an emergency in public health worldwide. Since this pandemic disease was reported by the first time in Wuhan, China, in December 2019, more than 2 million people have tested positive globally and almost 140 thousand have died [1, 2]. At this moment, the virus has been documented in every continent, with expressive outbreaks in Italy and New York. In Brazil, there are 291,579 confirmed cases and 18,859 deaths since the last report of the World Health Organization published in May 22nd, 2020 examining community transmission [3].

Scientific data regarding this new disease are rapidly accumulating [4–7]. Descriptions of epidemiological issues, incubation periods, pharmacological treatment, clinical course, risk factors, and several other evidences are being producing at the same time the world is struggling to minimize the damage. Early data about the general population suggest 80% of cases mild, 14% develop more severe symptoms, and 6% develop critical illness. The group most at risk includes individuals who are older than 50 years, those have chronic illnesses (i.e., heart disease and diabetes), and those who have chronic respiratory conditions (i.e. asthma) [7, 8]. Estimates show that one contagious person can infects between 2.2 and 2.4 other people [5]. Available data suggest the length of the incubation period of the virus is about 5 days, but it may range from 2 to 14 days [4].

Many questions are still being answered by researchers, including whether there is an effective pharmacological therapy to treat infected people [9]. The clinical outcomes associated with COVID-19 in pregnant women and babies are still uncertain. There is not enough data to confirm if the pregnant women are at a higher risk of deaths or respiratory complications associated with the virus, or if there they have an increased risk for premature delivery or other perinatal complications and vertical transmission. This is the third reported spillover of an animal coronavirus to human beings in the last two decades. Previous descriptions point to the severe acute respiratory syndrome (SARS) and the Middle East respiratory syndrome (MERS), diseases associated with severe perinatal outcomes, such as miscarriage, premature delivery, intrauterine growth restriction, and maternal death [10].

On the other hand, at this time, pregnant women are not considered a risk group for developing COVID-19 compared with the general population [10, 11]. But this information should be handled carefully because there are few epidemiological and clinical information for this group.

Researchers have been focusing on the technical-scientific mechanisms of the disease. In addition to the scientific community, the world population has been waiting for answers and remains alert and aware of the advances of the pandemic, with its routines altered or paralyzed to avoid contagion accelerated by human gatherings. People watch the news paralyzed as they see messages about the alarming number of people infected, the frequent deaths, and the collapse of the health system in some countries, overloaded by the number of serious cases that require hospitalization. There is some discussion arising regarding the panic spread by social media, through the dissemination of misinformation, conflicting information, and manipulated information [12–14].

The reactions are diverse: panic, anxiety, indifference, and the sense of awe at the extreme measures of social isolation required by the most world authorities. Emerging infectious diseases and pandemics may affect directly the women’s sexual and reproductive health and rights [15]. It is not yet known what impact this pandemic experience will have on the population’s mental and reproductive health, especially pregnant women, who will be the object of the study presented here.

There is a consensus that, during pregnancy, women are faced with major physical, psychological, and emotional changes, with ambiguous feelings, changing roles, doubts and, often, situations of vulnerability. The World Health Organization (WHO) has been pointing to the relevance of maternal mental health, indicating that around 10% of pregnant women and 13% of postpartum women in the world have some mental illness, primarily depression. In developing countries, these percentages may be even higher (15.6% during pregnancy and 19.8% after delivery). In addition to the risk of suicide, affected women may not function properly with regard to baby care and other activities, with consequences for the child’s overall and emotional development [16].

Pandemics like this are a source of anxiety, sadness, and fear. In addition to pregnancy and the uncertain scenario related to the disease, pregnant women infected with COVID-19 may be experiencing intense psychological suffering, which can cause serious consequences in terms of mental health. In this sense, understanding the experience of pregnant women infected by COVID-19 is an important issue for the development of intervention strategies for the promotion, prevention, and restoration of comprehensive health in this population. We aim to explore the emotional experiences of these pregnant women in regard to the illness process, their community relations, and the influence of social media.

## Method

**Fig. 1:**
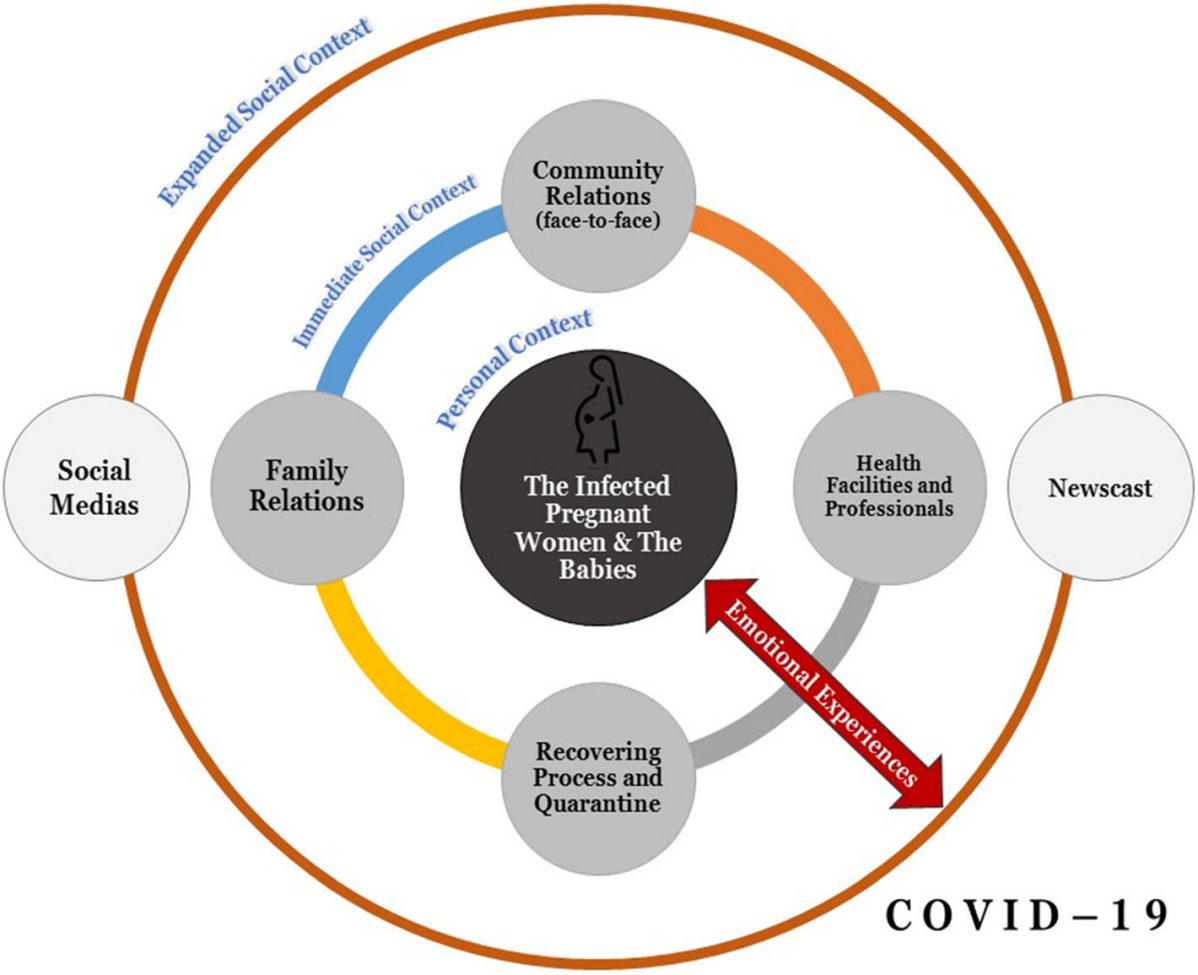
The theoretical model of the aims to be explored in the research

We aim to understand the experiences of women infected by the COVID-19 during pregnancy. We will explore their emotional perceptions concerning the illness process during the gestational period in regard their own and babies’ health, how they emotionally manage the recovery period, the treatment and the quarantine, and also the relationships with health services and professionals. We intend to understand the women’s perceptions about their family and community relationships after receiving a diagnosis, namely, their perceptions about the neighborhood, friends, co-workers, and other immediately related people. Finally, we propose to explore the influence of social media and newscasts on pregnant women’s feelings about the COVID-19 infection. Figure 1 demonstrates the theoretical model of the aims.

We will conduct a qualitative approach to achieve the aims of the study, using in-depth interviews and thematic analysis. This study protocol follows the criteria of the COREQ Checklist [17].

### Participants

We will include women infected by COVID-19 during the gestational period who are 18 years old or more. They must be emotionally and cognitively capable of verbally communicating in order to participate in the study and symptoms must be remitted at the time of data collection. As exclusion criteria, we will consider illiteracy, psychiatric/neurological or other illnesses that make it impossible to answer question, and participants who do not agree to have their interviews recorded. The purposive sampling will consider the concept of theoretical saturation, proposed by Glaser and Strauss [18], which means we will include new participants until data has reached sufficient consistency to meet the initial objectives.

### Setting

The participants will be recruited at a tertiary university hospital specializing in the women’s health. The hospital is from the public health system in the Brazilian Southeast. The hospital comprises more than 100 cities, containing 5 million people. It is a reference center for high-risk pregnancies. Since the first case notified in Brazil (February 27, 2020), the health team has been preparing to provide specialized assistance in the fight against the pandemic.

### Instruments

We will use three instruments for data collection: (1) Semi-Directed Interviews of Open and In-depth Questions; (2) Sociodemographic and Health Data Sheets; and (3) Field Diaries. The interview script will start with the trigger question: *“Tell me how you are experiencing this pregnancy”*. The complete list of questions designed for the study is shown in Table 1.

**Table 1:** The interview script

Open-ended and in-deep questions for the interviews	
***Trigger question: Tell me how you are/were experiencing this pregnancy.***	
How did you feel when you were diagnosed with COVID-19?	
Tell me about your experience with this disease before and after the infection	
How do you feel about your own health? What about the baby’s health?	
Tell me about your experience with your partner and / or your closest family about COVID.	
How has it been with other people close to you, such as friends, neighbors, co-workers?	
How did you feel about the care you received from the health team? What is your perception of the recovery period and treatment of the disease?	
How do you understand the quarantine or the social isolation? How was it for you?	
Do you think that the media influenced your feelings about COVID? For example, whatsapp messages and groups, newscasts, instagram, facebook, twitter.	

The Data Sheet will be self-referred or checked in the medical record. The sociodemographic information will include: age, schooling, religion, marital status, household composition, occupation/paid work, family income, and race. The health data will include symptoms of COVID-19 (fever, coryza, odynophagia, myalgia, diarrhea, meningoencephalitis, myocarditis, respiratory failure, others), symptom onset date, date when the pregnant woman first sought hospital admittance from symptoms of COVID-19, duration of the hospitalization and when needed, and resources and details of the medical treatment (e.g., drugs, artificial respirators). We will also collect obstetric information, including gestational age or delivery date, number of pregnancies, number of deliveries, history of miscarriages, maternal pathologies (i.e. diabetes, cardiopathy, HIV, others), and obstetrics pathologies (i.e. gestation diabetes, hypertension/ eclampsia, oligoamine, etc.).

The field diary is a systematic instrument of observation, in which the researcher examines the global behavior of the participant along with the interview. It contains non-verbal communication, including the personal presentation of the participant, her global behavior, body expressions, gestures, facial expressions, style and alterations in speech (silences, choking speech, lapses of the language, inhibited and uninhibited placements, changes in timbre and volume of voice), laughter, smiles, cries, and similar manifestations.

### Data collection procedures

Due to the social isolation in Brazil owing to COVID-19, every contact with the participants will be done virtually, through phone calls or videoconferences. The eligible women will be provided by one researcher (FGS), a medical doctor involved in person in the health assistance of the patients at the hospital. Another researcher (JVFJ)—a psychologist with experience in qualitative interviews and pregnancy—will conduct the data collection. Aiming to achieve a random sample, the interviewer will select the eligible patients from the listed names randomly.

First, eligible women will be contacted by telephone, and the researcher will invite them to participate in the study. The researcher will set out the procedures and terms of participation in the research. Thereafter, the researcher will send the consent form by e-mail or by a messaging app, according to the women’s preference, that must be answered by the participant if she agrees to participate. To aid in participant understanding, the researcher will send a video presenting the full consent term.

Data collection will be performed by videoconference or telephone call. We will audio record every interview. At the time of recording, participants will be invited to verbally offer their consent to participate in the study. Data from the patient’s medical record will be accessed later to verify information referring to the problems of contamination by COVID-19.

The virtual contact with the participant requires a particular attention on the appropriate *rapport* for the interview, especially regarding the environment around the women during data collection. We will recommend an appropriate, quiet, and private room where the participants may stay during the whole interview, so they will be able to express themselves more comfortably. The interviewer, on the same way, must assure confidentiality during data collection, using a private and quiet place to conduce the interviews. Moreover, it’s important to emphasize to them that we are not expecting a correct answer for the questions and we are interested on how the woman are truly feeling truly, with no judgment involved. After finishing the interview script, we will ask the participants if they want to say something else.

### Data analysis

We will transcribe verbatim the audio-recorded interviews. The concepts of medical psychology will be brought up for discussion. The data analysis will be conducted by two researchers independently (JVFJ and LR). We will discuss the categorization process with the co-authors and with the research team from the SARHAS Group (Research Group on Reproductive Health and Healthy Habits) that belongs to the School of Medical Sciences of the University of Campinas.

The data analysis will follow the thematic analysis, proposed by Nowell et al. [19], in which the textual data, produced from the transcription of the interviews, the field diaries, and the data sheet (when applicable), will go through a process of coding the raw data until reaching the consistency of themes. The step-by-step and description of each analysis phase is shown in Table 2. The NVIVO software will be used, which contributes to the organization and textual analysis through the simple frequency of words in the code formulation phase, validating the analyzes performed by the researchers.

**Table 2:** The Thematic Analysis

Phases of the Thematic Analysis	Description	Means of stablishing trustworthiness
**Phase 1:**	Phase in which the researchers are familiarized with the depth and breadth of the available data (interviews transcripts and field diary). The process of creating initial ideas about the data begins.	- Prolonged involvement with the data
**Familiarizing yourself with data**		- Triangulation of different types of qualitative data
		- Documentation of reflective and theoretical thoughts
		- Documentation of potential codes and themes
		- Storage of raw data in organized files
		- Keep records of field diaries, transcripts, and scientific articles
**Phase 2:**	Production of initial codes, a theorizing activity that requires researchers to revisit the data constantly. The codes will index important sections of the text, using software such as NVivo.	- Peer debriefing
**Generating initial codes**		- Reflective diary on data analysis
		- Structuring the coding process
		- Review of produced codes
		- Documentation of all meetings with researchers
**Phase 3:**	Production of themes that will unite the codes, bringing meaning and identity to the data according to the research question.	- Discussion or triangulation with researchers
**Searching for themes**		- Layout to give meaning to the text (use of maps, flowcharts, etc.)
		- Keep detailed notes on the development of hierarchical themes and concepts
**Phase 4:**	Refining the themes to check if there is a coherent standardization, according to the entire data. It is possible to return to the raw data and produce new codes that were not covered in the previous phases.	- Discussion or triangulation with researchers
**Reviewing themes**		- Approval of themes and sub-themes by team researchers
		- Referential suitability test through the return to raw data
**Phase 5:**	The researchers determine which aspect of the data each theme captures and identify what is interesting about it and why.	- Discussion or triangulation with researchers
**Defining and naming themes**		- Team consensus on topics
		- Documentation of team meetings on the topics
		- Documentation of the theme naming process
**Phase 6:**	When the themes are already defined, it is possible to complete the final analysis. Provide a concise, coherent, logical, non-repetitive, and interesting description of the data within and between themes	- Discussion with researchers
**Producing the report**		- Describe the coding and analysis process in sufficient detail
		- Detailed context descriptions
		- Description of the audit process
		- Report on the reasons for the theoretical, methodological and analytical choices throughout the study

### Trustworthiness and Limitations

We will consider the four strategies for ensuring trustworthiness: credibility, transferability, dependability, and confirmability [20]. We will also consider the COREQ Checklist for reporting qualitative research.

In regard the limitations of this study, we consider that every qualitative research project chooses a specific methodological model that offers a point of view among other possibilities of approaching the research questions. Moreover, a cross-sectional study takes place at a specific moment in the lives of the participants, in which conflicts may emerge in different psychological and relational points. However, the validity of the study is not compromised.

### Discontinuation and Ethical Issues

This study protocol received ethical approval from the local committee and follows the Declaration of Helsinki and the national legislation. Both the researcher and participant will be free to interrupt or suspend the interview in situations in which they perceive that the approach is negatively affecting the patients’ emotional state. Participants may withdraw from the study at any time, without justification, and without affecting the service they receive at the hospital.

## Discussion

This study will shine light on the lived experience of women infected by the COVID-19 during pregnancy. We have not found other studies adopting this approach, and the results will make valuable contributions to health care services and policy, especially for understanding the personal context, the immediate social context, and the expanded social context influencing the feelings of this women. This is important due to the relevance of the COVID-19 pandemic and may offer insights for health professionals and police makers.

## Data Availability

Not applicable.

## Abbreviations

SARS-CoV-2: Severe acute respiratory syndrome-novel coronavirus
COVID-19: Coronavirus
